# Artificial intelligence approaches for tinnitus diagnosis: leveraging high-frequency audiometry data for enhanced clinical predictions

**DOI:** 10.3389/frai.2024.1381455

**Published:** 2024-05-07

**Authors:** Seyed-Ali Sadegh-Zadeh, Alireza Soleimani Mamalo, Kaveh Kavianpour, Hamed Atashbar, Elham Heidari, Reza Hajizadeh, Amir Sam Roshani, Shima Habibzadeh, Shayan Saadat, Majid Behmanesh, Mozafar Saadat, Sahar Sayyadi Gargari

**Affiliations:** ^1^Department of Computing, School of Digital, Technologies and Arts, Staffordshire University, Stoke-on-Trent, United Kingdom; ^2^Student Research Committee, Urmia University of Medical Sciences, Urmia, Iran; ^3^Department of Computer Science and Mathematics, Amirkabir University of Technology, Tehran, Iran; ^4^Department of Cardiology, School of Medicine, Urmia University of Medical Sciences, Urmia, Iran; ^5^Department of Otorhinolaryngology - Head and Neck Surgery, Imam Khomeini University Hospital, Urmia, Iran; ^6^Department of Audiology, Tabriz University of Medical Sciences, Tabriz, Iran; ^7^Hull York Medical School, University of York, York, United Kingdom; ^8^Department of Mechanical Engineering, School of Engineering, University of Birmingham, Birmingham, United Kingdom

**Keywords:** tinnitus diagnosis, high-frequency audiometry, machine learning in audiology, point detection in audiometry, objective tinnitus assessment, advanced auditory data analysis

## Abstract

This research investigates the application of machine learning to improve the diagnosis of tinnitus using high-frequency audiometry data. A Logistic Regression (LR) model was developed alongside an Artificial Neural Network (ANN) and various baseline classifiers to identify the most effective approach for classifying tinnitus presence. The methodology encompassed data preprocessing, feature extraction focused on point detection, and rigorous model evaluation through performance metrics including accuracy, Area Under the ROC Curve (AUC), precision, recall, and F1 scores. The main findings reveal that the LR model, supported by the ANN, significantly outperformed other machine learning models, achieving an accuracy of 94.06%, an AUC of 97.06%, and high precision and recall scores. These results demonstrate the efficacy of the LR model and ANN in accurately diagnosing tinnitus, surpassing traditional diagnostic methods that rely on subjective assessments. The implications of this research are substantial for clinical audiology, suggesting that machine learning, particularly advanced models like ANNs, can provide a more objective and quantifiable tool for tinnitus diagnosis, especially when utilizing high-frequency audiometry data not typically assessed in standard hearing tests. The study underscores the potential for machine learning to facilitate earlier and more accurate tinnitus detection, which could lead to improved patient outcomes. Future work should aim to expand the dataset diversity, explore a broader range of algorithms, and conduct clinical trials to validate the models' practical utility. The research highlights the transformative potential of machine learning, including the LR model and ANN, in audiology, paving the way for advancements in the diagnosis and treatment of tinnitus.

## 1 Introduction

Tinnitus, commonly known as ringing in the ears, is a prevalent condition characterized by the perception of noise or ringing in the ears when no external sound is present (Roberts et al., 2010). This condition affects ~15–20% of people globally, making it a significant public health concern (Atik, 2014). The impact of tinnitus is multifaceted, ranging from minor annoyance to severe disruption in daily life, including difficulties with concentration, sleep disturbances, and even depression (De Ridder et al., 2021). The complexity of tinnitus, both in its manifestation and underlying causes, has posed a challenge for effective diagnosis and management (Cima et al., 2019). Traditional diagnostic methods, primarily based on patient self-report and basic audiometry, often fail to capture the nuanced nature of this condition, leading to a critical need for more sophisticated and accurate diagnostic tools.

Conventional audiometric tests typically assess hearing thresholds up to 8 kHz, which may not adequately represent the high-frequency hearing loss often associated with tinnitus (Kara et al., 2020). High-frequency audiometry, extending beyond this standard range, has shown promise in detecting subtle hearing anomalies potentially linked to tinnitus (Yildirim et al., 2010). However, the interpretation of high-frequency audiometry data is complex and requires a nuanced understanding of auditory function. The advent of machine learning offers a novel approach to interpret these intricate patterns, potentially leading to more accurate and earlier diagnosis of tinnitus. Therefore, there is a pressing need to explore and develop advanced diagnostic methods that utilize high-frequency audiometry data, augmented by machine learning techniques, to improve tinnitus diagnosis.

**Figure 1:**
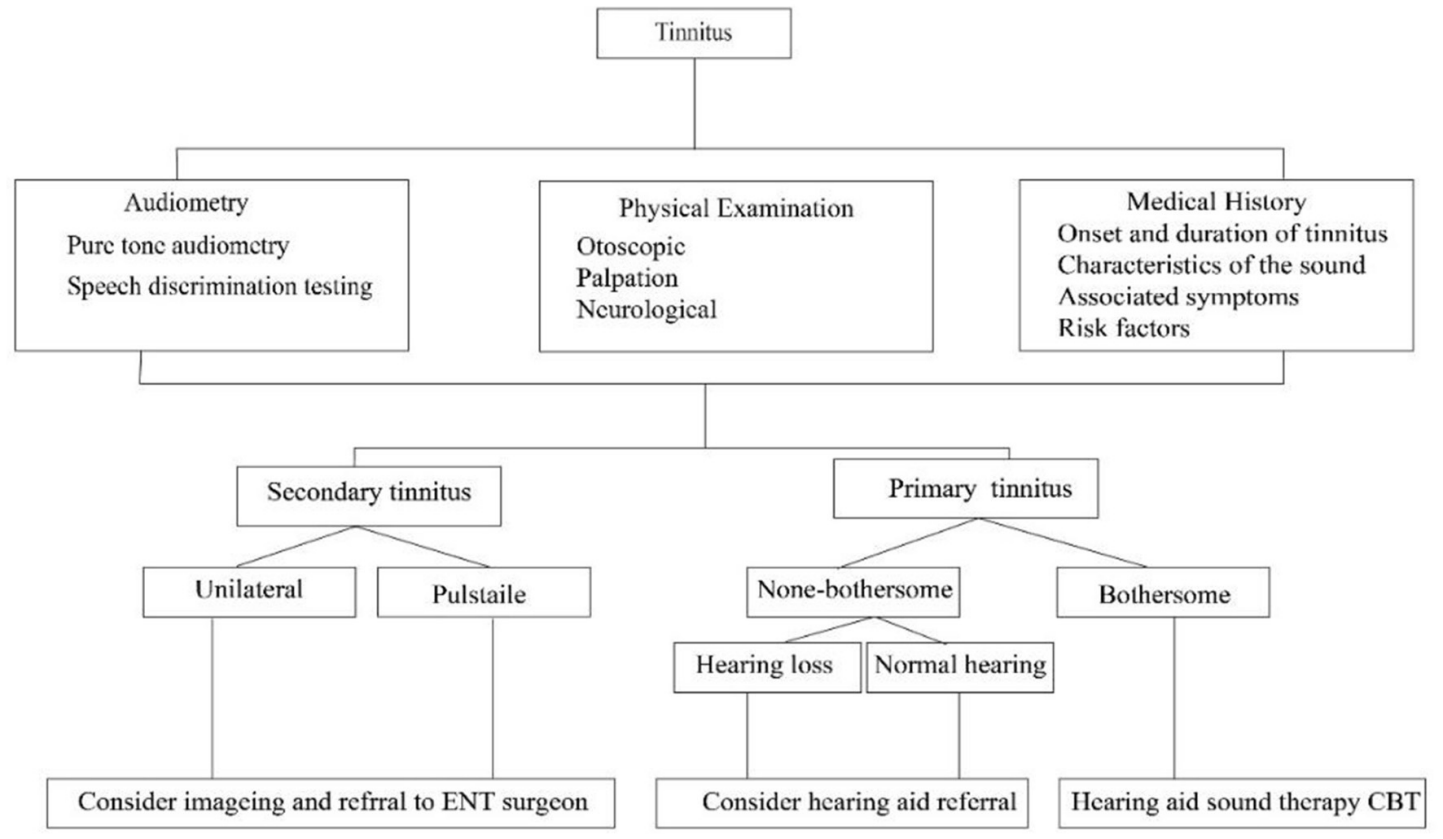
Flowchart of the Traditional Diagnostic Pathway for Tinnitus, delineating the evaluation process from initial assessment to specific management strategies based on the type and characteristics of tinnitus.

Figure 1 provides a flowchart depicting the traditional diagnostic pathway for tinnitus, starting with an initial assessment that includes Medical History, Physical Examination, and Audiometry. From here, the pathway bifurcates into secondary tinnitus and primary tinnitus. Secondary tinnitus further branches into “Unilateral”, where imaging and referral to an Ear, Nose, and Throat (ENT) surgeon are considered, and 'Pulsatile', which follows the same recommendation. For primary tinnitus, the pathway splits into “non-bothersome”, which may lead to a hearing aid referral if hearing loss is present, and 'Normal hearing', with no further action indicated. “Bothersome” primary tinnitus prompts consideration of a hearing aid, sound therapy, or Cognitive Behavioral Therapy (CBT), depending on whether the patient has concomitant hearing loss or not. This flowchart encapsulates the decision-making process in clinical settings for the management and treatment of tinnitus based on its characteristics and the patient's hearing profile.

This study aims to leverage machine learning algorithms to analyse high-frequency audiometry data for the effective diagnosis of tinnitus. By employing sophisticated data analysis techniques, the study seeks to uncover patterns in audiometry data that are indicative of tinnitus, which might be undetectable through traditional analysis methods. The primary objective is to develop a predictive model that can accurately classify individuals as having tinnitus based on their high-frequency audiometry results. This approach is anticipated to enhance the diagnostic process, offering a more objective and reliable means of identifying tinnitus, thereby facilitating timely and appropriate intervention.

**Figure 2:**
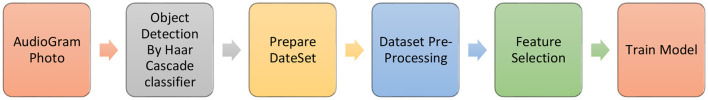
Study pipeline.

Figure 2 illustrates the streamlined workflow of our study. Initially, audiogram photos are processed using an Object Detection method powered by Haar Cascade classifiers, effectively identifying key features within the images. The dataset is then meticulously prepared, aligning the extracted data into a structured format. Subsequent dataset preprocessing ensures data quality and consistency, which is critical for accurate analysis. The workflow then transitions to feature selection, where the most significant variables, identified through rigorous statistical methods, are chosen for their predictive value. Finally, the selected features are used to train the machine learning model, culminating in a tool that can effectively differentiate between the presence and absence of tinnitus in individuals based on high-frequency audiometry data. This abstract encapsulates the methodical approach taken from data acquisition to the final model training, reflecting the robustness of our research methodology.

The paper is structured as follows: Section 2 presents a comprehensive literature review, discussing existing diagnostic methods for tinnitus and previous applications of machine learning in audiology. Section 3 describes the methodology, including data collection, preprocessing, feature engineering with a focus on point detection in high-frequency audiometry, model selection, and validation strategies. Section 4 details the results of the model, including its performance and the specific contribution of point detection techniques. Section 5 comprises a thorough discussion on the interpretation of the results, the significance of point detection, and a comparison with existing literature. The paper concludes with Section 6, summarizing the findings and implications for clinical practice, along with potential directions for future research.

## 2 Literature review

### 2.1 Current diagnostic methods for tinnitus

Tinnitus, a condition characterized by the perception of sound where no external source is present, presents a significant challenge in terms of diagnosis and treatment (Baguley et al., 2013). The current diagnostic methods for tinnitus are diverse, each with their own strengths and limitations. This section reviews the most prominent methods used in clinical practice and research. The most common method for diagnosing tinnitus involves subjective assessments, where patients describe their symptoms and answer questions about their condition. This includes various standardized questionnaires like the Tinnitus Handicap Inventory (THI) and the Tinnitus Functional Index (TFI) (Boecking et al., 2021). While these tools are valuable for understanding the impact of tinnitus on a patient's life, they rely heavily on self-reporting and are subjective in nature. They do not provide objective measures of tinnitus presence or severity (Fernández et al., 2023).

In the pursuit of enhancing diagnostic accuracy and efficiency in audiology, significant strides have been made in the realm of fast and efficient audiometry. Among the pioneering advancements, the work of Myburgh and Barbour (Twinomurinzi et al., 2024) stands out for their application of active transfer learning in audiogram estimation. They have demonstrated that active transfer learning can significantly expedite the process of audiogram estimation, thereby reducing the time required for audiometric evaluations without compromising accuracy. This approach not only aligns with the objectives of our research to leverage machine learning for enhanced clinical predictions but also highlights the potential for integrating such methodologies to refine the diagnostic process further. By applying transfer learning principles, we can potentially streamline the analysis of high-frequency audiometry data, paving the way for faster and more efficient diagnostic protocols. The incorporation of these references provides a broader perspective on the current state of technological advancements in audiometry. It underscores the relevance of exploring and integrating machine learning techniques, such as active transfer learning, to address the complexities of tinnitus diagnosis.

Pure-tone audiometry is a standard hearing test used to assess the degree and type of hearing loss, which can be associated with tinnitus. It involves the patient listening to a range of tones at various frequencies and volumes (Vielsmeier et al., 2015). Standard audiometry typically tests frequencies up to 8 kHz, which may not capture high-frequency hearing loss that is often linked with tinnitus (Masalski et al., 2018). Extending beyond the conventional range, high-frequency audiometry tests frequencies up to 16 kHz or higher (Shim et al., 2009). It is increasingly being recognized for its potential to detect early stages of hearing loss, especially in the high-frequency range, which might be related to tinnitus (Song et al., 2021). The interpretation of high-frequency audiometry data is more complex and is not yet widely adopted in standard clinical practice (Song et al., 2021).

Otoacoustic Emissions (OAEs) are sounds emitted by the inner ear when the cochlea is stimulated by a sound. Measuring these emissions can provide insights into the functioning of the cochlea, which may be related to tinnitus (Serra et al., 2015). OAEs are not specific to tinnitus and can be influenced by various factors, including middle ear conditions (Serra et al., 2015). Techniques like functional Magnetic Resonance Imaging (fMRI) and positron emission tomography (PET) have been used to study changes in brain activity in individuals with tinnitus (Eichhammer et al., 2007). These methods are more research-focused and are not practical for routine clinical diagnosis due to their cost, complexity, and availability.

Tests like auditory brainstem response (ABR) and electrocochleography (ECoG) assess the electrical activity in the auditory pathway and have been used in tinnitus research. These tests are more invasive and are generally used for research purposes rather than routine diagnosis (Park et al., 2023). While there are various methods available for diagnosing tinnitus, many of them have limitations in terms of objectivity, practicality, and sensitivity, particularly in detecting tinnitus associated with high-frequency hearing loss. This underscores the need for developing more advanced and precise diagnostic tools, such as the application of machine learning techniques to high-frequency audiometry data, to improve the accuracy and reliability of tinnitus diagnosis.

### 2.2 Machine learning applications in audiology

The application of machine learning in audiology has been a burgeoning field of research, offering new avenues for diagnosing and understanding various auditory conditions, including tinnitus. This section reviews the previous work done in the intersection of machine learning and audiology, focusing on how these advanced computational techniques have been employed to enhance auditory health care. Several studies have successfully employed machine learning algorithms to classify types and degrees of hearing loss. By analyzing audiometric data, machine learning models have been able to distinguish between normal hearing, sensorineural hearing loss, and conductive hearing loss with high accuracy. Commonly used techniques in these studies include Support Vector Machines (SVM) (Chen et al., 2018), Neural Networks No Matches Found, and Decision Trees (Lenatti et al., 2022).

Machine learning has played a significant role in improving speech recognition systems, particularly in challenging listening environments. This is crucial for individuals with hearing impairments, as background noise can significantly hinder speech comprehension (Zhang et al., 2018). Advanced algorithms such as recurrent deep neural networks (Weng et al., 2014) and Convolutional Neural Networks (CNNs) (Abdel-Hamid et al., 2014) have been utilized to enhance signal processing and speech intelligibility. Machine learning has been applied to optimize cochlear implant settings for individual usersNo Matches Found. Personalized machine learning models have shown potential in predicting the most effective implant configurations, enhancing speech perception for implant users. Techniques like Genetic Algorithms (Baskent et al., 2007) and Reinforcement Learning No Matches Foundhave been explored for this purpose.

Machine learning has been employed to analyse patterns in audiometric data and patient-reported outcomes to better understand and categorize tinnitus (Crowson et al., 2020a,b; Palacios et al., 2020). This includes identifying tinnitus subtypes and predicting treatment outcomes. Various machine learning models, including Random Forests (Bromis et al., 2022) and Gradient Boosting Machines (Allgaier et al., 2022), have been used for pattern recognition and prediction in tinnitus data. There is emerging research on the development of automated audiometry systems using machine learning. These systems aim to provide accurate hearing assessments without the need for extensive human oversight (Wasmann et al., 2022). Algorithms like SVM (Sankari et al., 2023) and Neural Networks (Charih et al., 2020) are being integrated into automated audiometry for real-time analysis and interpretation of audiometric data.

In recent years, researchers such as Casolani et al. (2024), Cox and de Vries (2021), and Twinomurinzi et al. (2024). Song et al. (2015) have made significant contributions to the field of fast and efficient audiometry. Casolani et al. (2024) have investigated the validity of high-frequency audiometry tools based on Bayesian learning, demonstrating their reliability, repeatability, and efficiency in clinical settings. Their work underscores the potential of Bayesian active learning algorithms to provide accurate and rapid assessments of hearing thresholds, offering valuable insights without imposing substantial time burdens on patients during their visits.

Cox and de Vries (2021) have explored probabilistic modeling approaches, particularly Gaussian process (GP) models, to enhance the efficiency of audiometry procedures. By utilizing GP mixture models conditioned on side-information about subjects, they have proposed methods to better capture the statistical properties of hearing thresholds among populations, leading to more accurate and efficient audiogram estimations. Their research highlights the importance of improving underlying models to optimize audiometry procedures and enhance predictive accuracies, ultimately facilitating better diagnosis and quantification of hearing loss.

Furthermore, Twinomurinzi et al. (2024), along with Myburgh, has demonstrated the potential of active transfer learning in speeding up audiogram estimation. Their work showcases innovative approaches to leverage transfer learning techniques for faster and more efficient audiometry, offering promising avenues for future advancements in the field. Collectively, the contributions of these researchers underscore the ongoing efforts to develop novel methods and technologies for fast, reliable, and efficient audiometry, ultimately improving clinical screening and treatment outcomes for individuals with hearing impairments.

The integration of machine learning into audiology has opened new possibilities for personalized care, early detection, and a better understanding of complex auditory conditions like tinnitus. The success of machine learning in these areas highlights its potential in transforming diagnostic and therapeutic approaches in audiology, making it a promising tool for future innovations.

### 2.3 Research gap identification

While the application of machine learning in audiology has shown promising results in various aspects, there remains a significant research gap, particularly in the utilization of high-frequency audiometry data for the diagnosis of tinnitus. This subsection identifies and discusses the specific research gaps that this research aims to address. Most machine learning studies in audiology have focused on standard audiometry ranges, speech processing, and cochlear implant optimization. High-frequency audiometry, which extends beyond the conventional frequency ranges, has not been extensively explored with machine learning techniques. There is a need for in-depth research on how machine learning can be applied to interpret high-frequency audiometry data, especially for conditions like tinnitus where high-frequency hearing loss is a key factor.

Tinnitus diagnosis predominantly relies on subjective assessments and questionnaires. Objective diagnostic methods, particularly using advanced data analysis techniques, are relatively underexplored. Developing an objective, machine learning-based diagnostic tool using high-frequency audiometry data could significantly enhance the accuracy and reliability of tinnitus diagnosis. The role of specific points or patterns within high-frequency audiometry data in diagnosing tinnitus is not well-studied. Existing machine learning models in audiology often overlook these finer details in the data. The integration of point detection methodologies within machine learning frameworks for analyzing high-frequency audiometry data is a novel approach. This research aims to investigate how these specific data points can improve the diagnosis of tinnitus.

There is a disconnect between the advanced data analysis capabilities offered by machine learning and their practical application in clinical settings for tinnitus diagnosis. This research seeks to bridge this research gap by developing a clinically applicable machine learning model that can be readily used by audiologists and healthcare professionals. The complexity and variability of tinnitus symptoms pose a challenge for understanding its underlying mechanisms and effective diagnosis. By applying machine learning to high-frequency audiometry data, this research aims to contribute to a deeper understanding of tinnitus, potentially uncovering new insights into its characteristics and diagnosis.

The proposed research is set to address these research gaps by leveraging machine learning techniques to analyse high-frequency audiometry data for the objective diagnosis of tinnitus. This approach has the potential to transform current diagnostic practices, offering a more precise, data-driven method for identifying tinnitus.

## 3 Methodology

### 3.1 Data collection

The data for this study was meticulously collected from high-frequency audiometry tests conducted at the audiology clinic of Imam Khomeini Hospital in Urmia, Iran. Given the retrospective nature of our analysis, no consent forms were procured for the collection of data. This dataset plays a pivotal role in our research, providing the foundational information required for applying machine learning techniques to diagnose tinnitus. The specifics of the data collection process are outlined below:

#### 3.1.1 Participant selection

The study focused on two distinct groups for comparative analysis: the Tinnitus Group and the Control Group. The Tinnitus Group consisted of individuals who visited the clinic with tinnitus complaints and were included in the study only if their audiograms showed abnormalities, ensuring that the data represented genuine cases of auditory impairment commonly associated with tinnitus. Conversely, the Control Group was comprised of individuals who came for routine hearing check-ups without any tinnitus complaints. This group included only participants whose audiograms fell within the normal hearing range, providing a baseline for comparison with the Tinnitus Group.

#### 3.1.2 Data volume

The study incorporated a total of 509 audiograms. This comprised 242 audiograms from patients with tinnitus (exhibiting abnormalities) and 267 audiograms from the control group (with normal hearing).

#### 3.1.3 High-frequency audiometry tests

In this study, high-frequency audiometry was utilized, extending the test range beyond the standard limit of 8 kHz, which is typical in conventional audiometry. This approach allowed for a more in-depth analysis of the participants' hearing capabilities, especially in the higher frequency spectrum. The audiograms generated from this process provided a comprehensive dataset, featuring detailed threshold levels at various frequencies, thereby offering a thorough understanding of auditory functions across an expanded range.

#### 3.1.4 Ethical considerations

The study was conducted following strict ethical guidelines and was approved by the Ethics Committee of Urmia University of Medical Sciences, Iran, under the ethical code IR.UMSU.REC.1402.284. All data was anonymized and handled in compliance with privacy and ethical standards to protect participant confidentiality.

This dataset, encompassing a wide range of high-frequency audiometry results from both tinnitus sufferers and individuals with normal hearing, provides a unique opportunity to apply and evaluate machine learning models in the context of tinnitus diagnosis. The richness and diversity of the data are key to developing a robust and accurate diagnostic tool.

### 3.2 Data preprocessing

Data preprocessing is a crucial step in any machine learning project, as it involves preparing and cleaning the data to ensure that the machine learning algorithms can effectively process and analyse it (Sadegh-Zadeh et al., 2022a,b, 2023a,b). For this study, focused on diagnosing tinnitus using high-frequency audiometry data, the preprocessing involved several specific steps, particularly due to the complexity of extracting data from audiograms in PDF format. The following subsections detail the preprocessing steps taken.

In addressing the challenge of missing data within the dataset, a strategic approach was undertaken to ensure the integrity of the analysis and the reliability of our findings. Recognizing the varied nature of our data, encompassing both binary and continuous variables, the Multivariate Imputation by Chained Equations (MICE) method was employed. This choice was motivated by MICE's capability to handle different data types effectively through a flexible regression-based imputation model. For binary variables, imputation was conducted using logistic regression models within the MICE framework, allowing for the probabilistic estimation of missing values based on observable data. This ensures that the binary characteristics of these variables are preserved and accurately reflected in the imputed datasets.

Continuous variables were treated with predictive mean matching, a technique that identifies close matches for missing values from the pool of observed values, based on other variables in the dataset. This method is particularly suitable for our dataset as it maintains the original distribution of continuous variables without assuming normality. The implementation of the MICE method was facilitated by the “mice” package in R, chosen for its comprehensive functionality and flexibility in specifying imputation models tailored to the diverse range of variables in our study. Parameters and options used in the ' mice' package, including the number of imputations and iterations, were carefully selected to align with the nature of our data and the objectives of our analysis.

#### 3.2.1 Extraction of audiograms from PDF files

Each patient's audiometry data was stored in PDF files, which included audiograms for both the left and right ears. The first step involved extracting these audiograms from the PDF files. Due to the format of the data, conventional text extraction methods were not sufficient. Instead, image extraction techniques were employed to accurately retrieve the audiograms.

#### 3.2.2 Handling overlapping symbols in audiograms

Audiograms typically display both bone conductive and air conductive hearing thresholds, often represented by different symbols. In many cases, these symbols overlapped, creating challenges in accurately extracting the data points. To address this, advanced image processing techniques were employed. These included techniques like symbol recognition and separation algorithms to differentiate and accurately extract both types of hearing threshold data from the overlapping symbols.

#### 3.2.3 Normalization of audiogram data

Once extracted, the audiogram data was normalized to ensure consistency across all samples. This involved standardizing the range and scale of the frequency and decibel values. Normalization was essential to compare audiograms from different patients accurately and to prepare the data for effective machine learning analysis.

#### 3.2.4 Conversion to structured data format

The extracted and normalized audiogram data was then converted into a structured data format, such as CSV and a DataFrame, for ease of use in machine learning algorithms. Care was taken to maintain the integrity of the data during this conversion, ensuring that all relevant information, such as frequency and threshold levels, was accurately represented.

#### 3.2.5 Identification and handling of missing or anomalous data

The preprocessing phase also involved identifying any missing or anomalous data points within the audiograms. Depending on the nature and extent of the missing data, appropriate methods like data imputation or exclusion of incomplete records were used.

#### 3.2.6 Point detection preparation

Given the focus on point detection in high-frequency audiometry data, special attention was given to preparing the data for this analysis. This involved enhancing the resolution of the data points in the high-frequency range and applying preliminary filters to identify potential points of interest for further analysis.

Through these preprocessing steps, the audiometry data was transformed into a clean, structured, and analysis-ready format. This meticulous preparation was foundational to the subsequent application of machine learning algorithms for the accurate diagnosis of tinnitus.

### 3.3 Feature engineering

Feature engineering is a critical step in developing a machine learning model, as it involves selecting and transforming raw data into features that effectively represent the underlying problem to be solvedNo Matches Found. In the context of diagnosing tinnitus using high-frequency audiometry data, several specific features were extracted, focusing particularly on point detection techniques.

#### 3.3.1 Point detection in high-frequency audiometry

The point detection process in high-frequency audiometry plays a pivotal role in our methodology. It is designed to identify and analyse specific points in the audiogram that are crucial for diagnosing tinnitus. The following describes the methods and rationale for point detection in our dataset:

1. Use of Haar Cascade classifier for point extraction

The Haar Cascade classifier, a machine learning object detection algorithm, was utilized to extract points from the audiogram charts. Initially, various forms of points on the audiogram were identified and fed into the Haar Cascade model. These forms vary significantly between the right and left ears, necessitating the training of separate models for each. The Haar Cascade classifier is known for its efficiency in detecting objects in images. It requires examples set in a similar context to the target object but not the exact object itself. Therefore, when focusing on points for the left ear, right ear audiograms were used as negative examples in the model, and vice versa. This approach enhances the classifier's ability to differentiate relevant points specific to each ear.

2. Identification of key points in audiograms

Once the model was trained, it was employed to separately identify points for the right and left ears. The coordinates of these detected points in the image were then used. An internal scaling system within the audiograms was utilized to translate these coordinates into corresponding decibel levels and frequencies for each point in the audiogram. The accurate detection and interpretation of these points are crucial, as they represent the specific hearing thresholds that are essential for diagnosing tinnitus.

3. Extracted features

**Standard deviation of points (Std)**

Since tinnitus often manifests as one or several points that differ from others in an audiogram, the standard deviation of these points was calculated and used as a feature. This metric helps in identifying the variability and dispersion in the hearing thresholds. Figure 2 displays a box plot comparing the standard deviation (std) of high-frequency audiometry readings between two groups: “Normal” and “Tinnitus”. The “Normal” group, represented in blue, exhibits a narrower interquartile range (IQR) and a lower median, suggesting less variability in audiometry readings within this group. Conversely, the “Tinnitus” group, shown in orange, has a broader IQR and a higher median, indicating greater variability among individuals with tinnitus. The presence of outliers, depicted by diamonds, particularly in the “Tinnitus” group, suggests that some individuals with tinnitus have audiometry readings with a substantially higher standard deviation than the general tinnitus population. This visual suggests that there are notable differences in the variability of high-frequency audiometry readings between normal hearing individuals and those with tinnitus.

**Presence of points above 30 dB**

**Figure 3:**
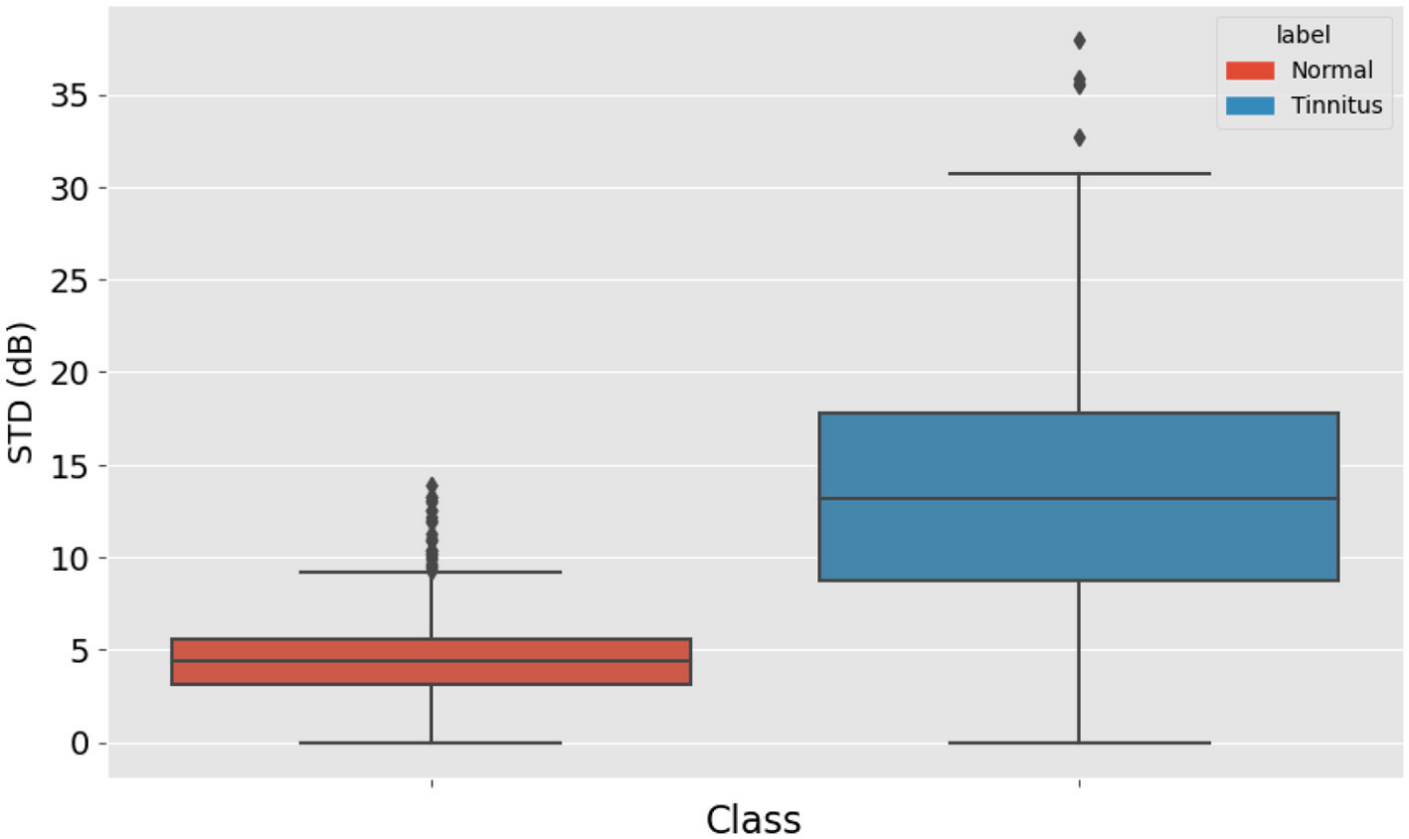
Box Plot of High-Frequency Audiometry Reading Variability, comparing the standard deviation in audiometry readings between normal hearing individuals and patients with tinnitus.

In tinnitus, certain indicative points in the audiogram are typically above 30 dB. The presence of such points significantly increases the likelihood of tinnitus, making this a valuable feature. Figure 3 presents a comparison between two groups, “Normal” and “Tinnitus”, based on a threshold value—denoted here as “Has Bigger than 30”. The “False” category represents cases where audiometry readings are below the threshold of 30, and “True” indicates readings above this threshold. In the “Normal” group, a vast majority fall into the “False” category, suggesting that normal hearing rarely exhibits values above this threshold. In contrast, for the “Tinnitus” group, there is a predominant count in the “True” category, indicating that readings exceeding the threshold are common among those with tinnitus. This distinction suggests that the threshold of 30 may be a significant indicator differentiating normal hearing from those with tinnitus, potentially serving as a diagnostic criterion in the assessment of tinnitus through audiometry.

**Average of points**

**Figure 4:**
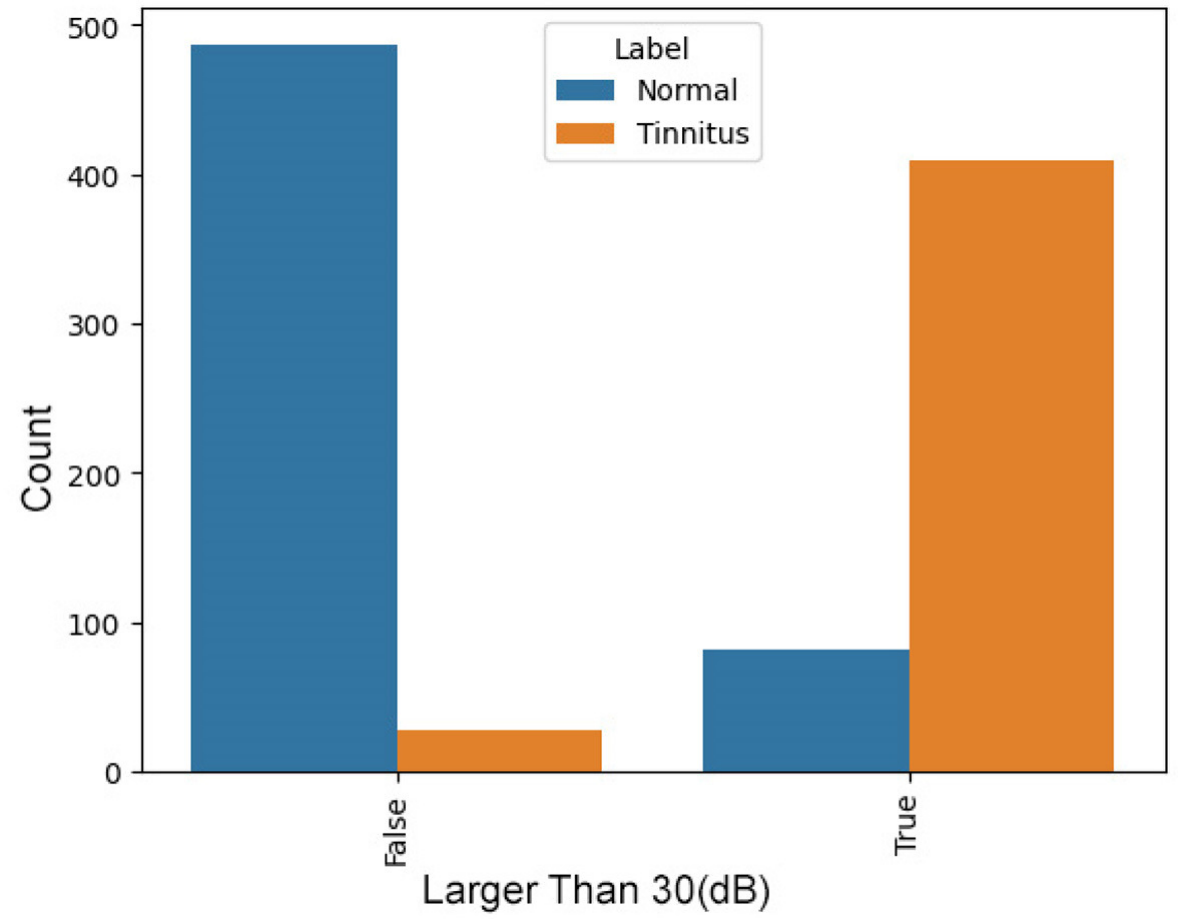
Distribution of Audiometry Readings Relative to a Threshold Value, illustrating the count of individuals with normal hearing and tinnitus patients based on whether their audiometry readings exceed a value of 30.

The average decibel level of all the points was also calculated. This average provides an overall sense of the hearing threshold pattern in the audiogram, contributing to the diagnosis. Figure 4 displays the distribution of mean high-frequency audiometry readings for two groups labeled “Normal” and “Tinnitus”. The “Normal” group's box plot, colored in blue, has a relatively low median value and a tight IQR, indicating little variation around the mean audiometry reading for this group. The “Tinnitus” group, shown in orange, exhibits a higher median value and a broader IQR, suggesting a higher mean audiometry reading with more variability among patients with tinnitus. Additionally, the “Tinnitus” group has several outliers above the upper whisker, indicating that some tinnitus patients have mean audiometry readings significantly higher than the general tinnitus population. This visualization suggests that mean audiometry readings are generally higher and more varied in the tinnitus group compared to the normal group, which could be indicative of the auditory profile associated with tinnitus.

The feature engineering process, especially the incorporation of point detection techniques, is fundamental to our approach. It allows the machine learning model to accurately interpret the high-frequency audiometry data, thereby enhancing the precision of tinnitus diagnosis.

### 3.4 Model selection and implementation details

This section includes detailed implementation details of the LR model used in our study. These additions aim to provide clarity and facilitate reproducibility of our findings.

#### 3.4.1 Feature selection

The LR model was developed using a comprehensive set of features extracted from high-frequency audiometry data. Key features included the average threshold at high frequencies (8 kHz−16 kHz), the standard deviation of thresholds across these frequencies, and specific point detection markers identified as significant for tinnitus presence. The selection of these features was informed by prior literature on audiological markers for tinnitus and a preliminary analysis showing their correlation with tinnitus diagnosis.

#### 3.4.2 Model configuration

We employed a LR model with L2 regularization to prevent overfitting, given the high dimensionality of our feature space. The regularization strength (C) was set to 1.0 after evaluation of model performance across a range of values from 0.01 to 100, using a 5-fold cross-validation on the training dataset. This approach balances model complexity with prediction accuracy, ensuring generalizability.

#### 3.4.3 Hyperparameter settings

The LR model's hyperparameters were meticulously selected to optimize performance. The primary hyperparameter, the regularization strength **C**, was determined through a grid search within the aforementioned range. The grid search evaluated the model's Area Under the Receiver Operating Characteristic Curve (AUC) on a validation set, identifying the optimal **C** value that maximizes AUC while maintaining model simplicity.

#### 3.4.4 Evaluation techniques

Model performance was assessed using several metrics: accuracy, precision, recall, F1 score, and the Area Under the Receiver Operating Characteristic Curve (AUC-ROC). We utilized stratified 5-fold cross-validation to ensure a thorough evaluation, accounting for the imbalance in our dataset between tinnitus and non-tinnitus samples. This technique provided a robust estimate of the model's ability to generalize to unseen data.

In the pursuit of developing a robust model for the diagnosis of tinnitus using high-frequency audiometry data, the choice of an appropriate machine learning model is critical. Given the observed significant and meaningful relationships between the features and the target, LR has been identified as a suitable starting point for model development.

#### 3.4.5 Advantages of logistic regression

LR is relatively straightforward and offers interpretable results, making it an ideal choice for initial model development (Menard, 2010). This is especially important in clinical applications where understanding and explaining the model's decision-making process is as crucial as its predictive power (Zabor et al., 2022). As tinnitus diagnosis typically involves a binary outcome (presence or absence of tinnitus), LR is well-suited for this task due to its efficiency in modeling binary dependent variables (Stoltzfus, 2011). The decision to use LR is further supported by the linear relationships identified between the features (audiometry data) and the target (tinnitus diagnosis). LR performs well when there is a linear relationship between the independent variables and the log odds of the dependent variable (Miguel-Hurtado et al., 2016).

### 3.5 Model training and validation

The training and validation of the LR model are meticulously structured to ensure reliability and robustness in the diagnosis of tinnitus. The dataset was split into two parts: 70% of the data was used for training the model, and the remaining 30% was reserved for testing. This split aims to provide a substantial amount of data for the model to learn from, while still retaining a significant portion for an unbiased evaluation of its performance.

It is crucial to note that tinnitus is often defined at the level of each ear. Therefore, the data for the left and right ears were labeled and analyzed separately. This approach acknowledges the potential differences in tinnitus manifestation between ears and ensures that the model is trained and evaluated on data that accurately reflects this clinical reality. The LR model was trained on the labeled data, learning to discern patterns and relationships indicative of tinnitus. The model's parameters were adjusted during training to minimize errors and improve its predictive accuracy.

To ensure that the model's performance is not specific to a particular subset of data, cross-validation was employed. This technique involves dividing the training data into several subsets, training the model on some subsets and validating it on the others. This process is repeated multiple times to ascertain the model's reliability and robustness. The model's performance was evaluated using metrics such as accuracy, precision, recall, and the AUC-ROC. These metrics provide a comprehensive understanding of the model's effectiveness in diagnosing tinnitus.

Through careful model selection and a rigorous training and validation process, the study aims to develop a reliable and clinically applicable machine learning model for tinnitus diagnosis using high-frequency audiometry data.

## 4 Results

### 4.1 Model performance

As shown in Table 1 the LR model's performance was quantitatively assessed using a confusion matrix and a classification report, which includes precision, recall, f1-score, and support for both “Normal” and “Tinnitus” classes. The overall accuracy of the model stood at 92%, with the following detailed metrics observed.

- **Confusion matrix**

- The confusion matrix displayed two rows and two columns, with the top row corresponding to the 'Normal' condition and the bottom row to 'Tinnitus'. There were 162 true positives where the model correctly identified the normal condition, and 118 true positives for correctly identified tinnitus cases. False positives were low, with 9 cases wrongly identified as tinnitus, and the model incorrectly classified 14 cases of tinnitus as normal. This matrix is indicative of a high true positive rate and a low false positive rate.

- **Classification report**

- **Normal condition**

- **Precision:** The model had a precision of 0.92 for the normal condition, meaning that 92% of instances predicted as normal were correct.
- **Recall:** The recall for the normal condition was 0.95, signifying that the model correctly identified 95% of all actual normal conditions.
- **F1-Score:** The f1-score for the normal condition was 0.93, reflecting a balanced precision-recall for this class.
- **Support:** The support for the normal condition was 171, indicating the number of true instances for the normal condition in the test set.

- **Tinnitus condition**

- **Precision:** Precision for tinnitus was slightly higher at 0.93, showing that when the model predicted tinnitus, it was correct 93% of the time.
- **Recall:** The recall for tinnitus was 0.89, indicating that the model identified 89% of all actual cases of tinnitus.
- **F1-Score:** The f1-score for the tinnitus condition was 0.91, also indicating a strong precision-recall balance for this class.
- **Support:** There were 132 instances of the tinnitus condition in the test set, as shown by the support value.

- **Overall metrics**

- The macro average and weighted average for precision, recall, and f1-score were all consistent at 0.92. The macro average provides an unweighted mean of the metrics for each class, while the weighted average takes into account the support for each class, giving a metric more reflective of the model's performance across the imbalanced dataset.

**Table 1:** LR Model Performance Metrics, displaying **(A)**. the confusion matrix with true and false positives and negatives, alongside**(B)**. precision, recall, f1-score, and support for the diagnosis of tinnitus vs. normal hearing conditions.

**A**				
**Class designation**			**Actual class**	
			**Positive**	**Negative**
Predicted classes		True	162	9
		False	14	118
**B**				
	**Precision**	**Recall**	**F1-Score**	**Support**
Normal	0.92	0.95	0.93	171
Tinnitus	0.93	0.89	0.91	132
Accuracy	-	-	0.92	303
Macro average	0.92	0.92	0.92	303
Weighted average	0.92	0.92	0.92	303

The high recall of 91% indicates the model's strong ability to identify true cases of tinnitus, which is critical in a clinical diagnostic tool to minimize the risk of overlooking affected patients. The balance of precision and recall across both classes, as represented by the f1-score, along with the high overall accuracy, suggests that the model is well-calibrated and performs robustly in differentiating between normal hearing conditions and tinnitus. This performance demonstrates the potential of machine learning approaches in augmenting diagnostic capabilities in the field of audiology, specifically for the condition of tinnitus.

### 4.2 Comparison with baselines

The performance of the LR model was compared against a range of baseline and advanced machine learning classifiers to evaluate its relative effectiveness in diagnosing tinnitus using high-frequency audiometry data.

The Table 2 summarizes the performance metrics for each model:

- **Logistic Regression (LR):** demonstrated excellent performance with an accuracy of 91.76%, an AUC of 97.06%, and a high recall and precision of 91.76% and 92.04% respectively, which are critical in clinical diagnostics. The F1 score of 91.71% and a moderate training time (TT) of 1.742 seconds indicate a well-balanced model with both high precision and recall.
- **K Neighbors Classifier (KNN):** exhibited comparable performance with an accuracy of 91.06%, although with a slightly lower recall and F1 score. Its AUC was impressive at 95.94%, suggesting good model discrimination capacity.
- **SVM—Linear Kernel:** the linear SVM model achieved a high precision of 91.15%, but its AUC is recorded at 0.00, which suggests there might be an issue with the calculation or interpretation of this metric for this model type.
- **Ridge Classifier:** showed lower performance metrics across the board with an accuracy of 89.92%. Similar to SVM, its AUC is 0.00, which may indicate a potential issue with the metric calculation.
- **Extra Trees Classifier:** this model had a significantly lower accuracy of 56.82% and an AUC of 68.11%, suggesting that it is not as effective in the given diagnostic context.
- **Naive Bayes, Decision Tree, Random Forest, Ada Boost, Gradient Boosting, LDA, LightGBM, CatBoost:** these models all shared an accuracy of 56.54%, with varying degrees of AUC, recall, precision, and F1 scores, none of which approached the effectiveness of the top-performing models.
- **Dummy Classifier:** as expected, the dummy classifier, which makes predictions based on simple heuristics, showed the lowest accuracy of 56.54% and an AUC of 50.00%, serving as a baseline indicator that any model performing similarly to this is performing no better than random guessing.
- **Quadratic Discriminant Analysis (QDA):** recorded the lowest accuracy of 49.18% and an AUC of 50.00%, indicating poor performance in this application.

**Table 2:** Performance metrics of various machine learning models for tinnitus diagnosis using high-frequency audiometry data, highlighting accuracy, AUC, recall, precision, F1 score, Kappa, MCC, and training time.

**Model**	**Accuracy**	**AUC**	**Recall**	**Prec**.	**F1**	**Kappa**	**MCC**	**TT (Sec)**
Logistic Regression	0.9176	0.9706	0.9176	0.9204	0.9171	0.8313	0.8346	1.720
K Neighbors Classifier	0.9106	0.9594	0.9106	0.9135	0.9099	0.8164	0.8202	0.036
SVM—Linear Kernel	0.9063	0.0000	0.9063	0.9115	0.9060	0.8096	0.8148	0.033
Ridge Classifier	0.8992	0.0000	0.8992	0.9082	0.8993	0.7978	0.8054	0.029
Extra Trees Classifier	0.5696	0.6811	0.5696	0.3647	0.4172	0.0108	0.0239	0.106
Naive Bayes	0.5654	0.5000	0.5654	0.3197	0.4084	0.0000	0.0000	0.027
Decision Tree Classifier	0.5654	0.5000	0.5654	0.3197	0.4084	0.0000	0.0000	0.027
Random Forest Classifier	0.5654	0.9404	0.5654	0.3197	0.4084	0.0000	0.0000	0.109
Ada Boost Classifier	0.5654	0.5000	0.5654	0.3197	0.4084	0.0000	0.0000	0.028
Gradient Boosting Classifier	0.5654	0.4365	0.5654	0.3197	0.4084	0.0000	0.0000	0.055
Linear Discriminant Analysis	0.5654	0.5000	0.5654	0.3197	0.4084	0.0000	0.0000	0.028
Light Gradient Boosting Machine	0.5654	0.9317	0.5654	0.3197	0.4084	0.0000	0.0000	0.075
CatBoost Classifier	0.5654	0.9677	0.5654	0.3197	0.4084	0.0000	0.0000	1.061
Dummy Classifier	0.5654	0.5000	0.5654	0.3197	0.4084	0.0000	0.0000	0.032
Quadratic Discriminant Analysis	0.4918	0.5000	0.4918	0.2461	0.3268	0.0000	0.0000	0.030

The LR model outperformed the majority of the compared models in terms of accuracy, AUC, recall, precision, and F1 score. The high AUC value is particularly noteworthy, as it suggests the model's strong capability to distinguish between the positive (tinnitus) and negative (normal) classes. Additionally, the F1 score, which is the harmonic mean of precision and recall, further indicates the model's balanced performance. The training time for the LR model was reasonable, suggesting that it is also a practical option for clinical settings where rapid diagnosis may be required.

**Figure 5:**
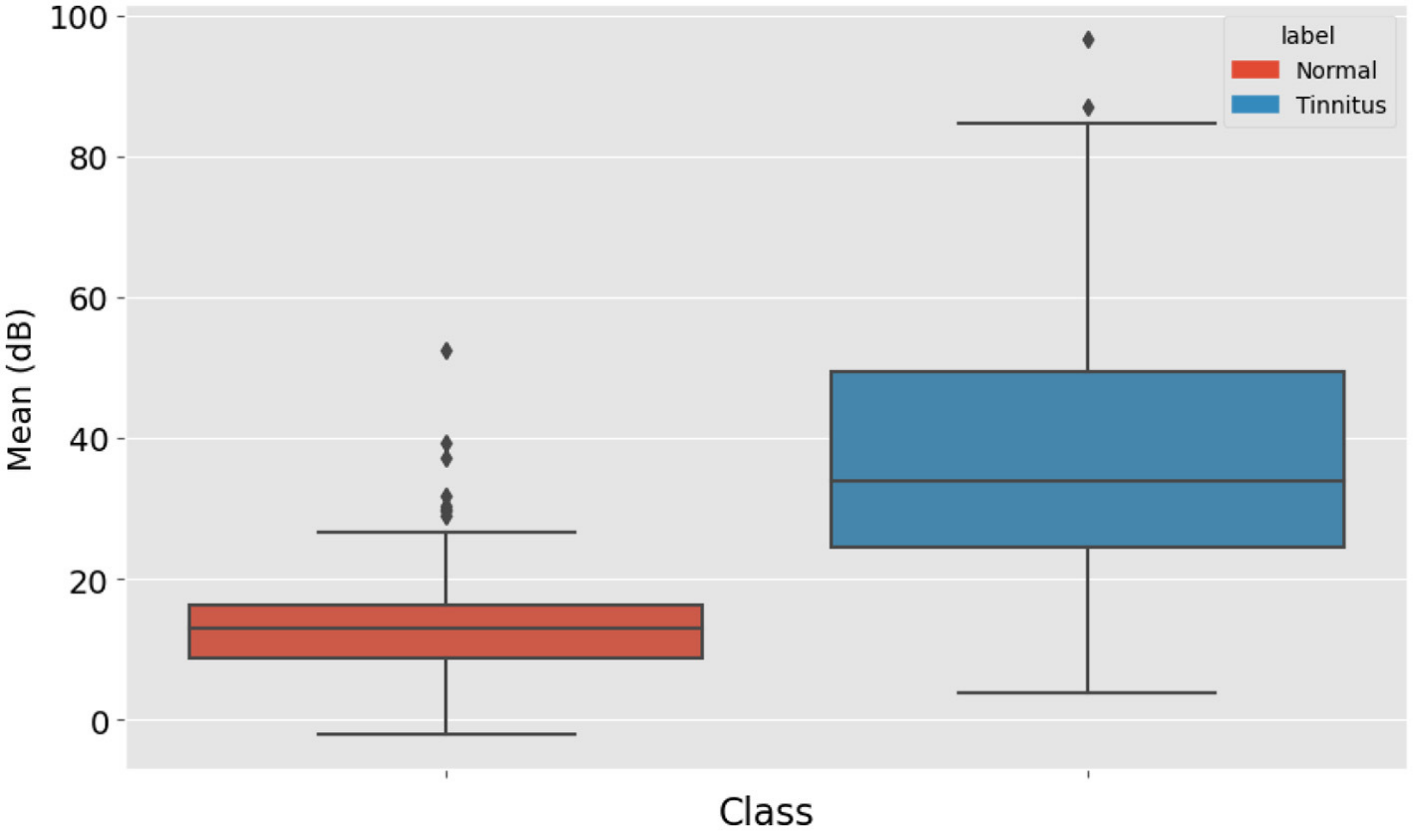
Box Plot Comparison of Mean High-Frequency Audiometry Readings between Normal Hearing Individuals and Tinnitus Patients, indicating higher and more variable readings within the tinnitus group.

Figure 5 illustrates ROC curves for various machine-learning models used in the diagnosis of tinnitus. Each curve represents the trade-off between the True Positive Rate (sensitivity) and False Positive Rate (specificity) for the respective models at different thresholds. Logistic Regression, Random Forest, Support Vector Machine, K Neighbors Classifier, Naive Bayes, Gradient Boosting Classifier, and QDA all show excellent performance with AUC scores close to or above 0.97, indicative of a high ability to distinguish between the tinnitus and non-tinnitus conditions. The Dummy Classifier serves as a baseline with an AUC of 0.50, equivalent to random guessing, underscoring the superior predictive capability of the other models. Notably, the Support Vector Machine and Naive Bayes exhibit slightly superior AUC scores at 0.98, suggesting a marginally better discrimination capacity. The ROC curves collectively demonstrate the efficacy of these models, with LR proving to be among the best performing in this specific application.

The LR model shows superior performance metrics compared to the other machine learning models tested, affirming its suitability for diagnosing tinnitus with high-frequency audiometry data. The results underscore the potential of LR as a robust, efficient, and accurate classifier for this clinical application.

In the analysis presented in Figure 5, the Extra Trees and Naive Bayes models demonstrated notably high AUC scores of 0.96 and 0.98, respectively. This outcome may initially appear surprising, given the complex nature of tinnitus diagnosis through high-frequency audiometry data. To clarify, these results can be attributed to several key factors specific to the preprocessing and analysis methodologies employed in this study.

Dataset Preparation and Feature Engineering:

The data preprocessing steps, including feature selection and normalization, were meticulously designed to reduce noise and highlight features most relevant to tinnitus diagnosis. This rigorous preprocessing may have enhanced the performance of models like Extra Trees and Naive Bayes, which benefit from clear, well-defined feature sets.

Ensemble and Independence Assumptions:

- **Extra Trees Classifier:** This model utilizes an ensemble of decision trees to create a robust classifier. The ensemble approach effectively leverages the diversity within the high-frequency audiometry data, allowing for a more accurate aggregation of predictions. This method is particularly adept at identifying complex, non-linear patterns across a range of frequencies, which is crucial in diagnosing conditions like tinnitus.
- **Naive Bayes Classifier:** Despite its simplicity, the Naive Bayes classifier excels in scenarios where features contribute independently to the outcome. In the context of our study, the assumption that each frequency band's threshold level independently influences the diagnosis of tinnitus aligns well with the Naive Bayes approach, leading to its surprisingly high AUC score.

Beyond AUC—Evaluating Model Performance:

It's crucial to note that while the AUC provides a useful measure of a model's ability to distinguish between classes, it does not encapsulate all aspects of model performance. Other metrics such as precision, recall, specificity, and the F1 score also play essential roles in evaluating the practical applicability of each model. As such, the high AUC scores observed for the Extra Trees and Naive Bayes models should be interpreted with consideration to their performance across these other metrics, particularly in a clinical decision-making context.

### 4.3 Performance of the artificial neural network (ANN) model

To compare classical machine learning models with deep learning approaches, a simple ANN was constructed and applied to the data. The ANN consisted of an input layer followed by three hidden layers. The activation function for the first two hidden layers was the Rectified Linear Unit (ReLU), and for the last layer, the sigmoid function was employed.

The model was compiled using binary cross entropy as the loss function and the Adam optimizer. After compiling, the model was fitted to the data with 100 epochs and a batch size of 32. The ANN achieved an accuracy of 94.06% (see Table 3). Here are the details of the ANN model's performance:

- **Precision:** The model exhibited high precision with 0.93 for class 0 (Normal) and 0.94 for class 1 (Tinnitus), indicating a high likelihood that the model's predictions are correct.
- **Recall:** The recall was 0.96 for class normal and 0.91 for class tinnitus, showing that the model has a high ability to detect the relevant class.
- **F1-Score:** The F1-scores were 0.94 for normal and 0.92 for tinnitus, suggesting a well-balanced model in terms of precision and recall.
- **Support:** The support, or the number of true instances for each class, was 114 for class normal and 88 for class tinnitus.

**Table 3:** **(A)** Confusion matrix of the ANN Model for Tinnitus Diagnosis, detailing, **(B)** precision, recall, f1-score, and support for each class.

**A**				
**Class designation**			**Actual class**	
			**Positive**	**Negative**
Predicted classes		True	109	5
		False	8	80
**B**				
	**Precision**	**Recall**	**F1-Score**	**Support**
Normal	0.93	0.96	0.94	114
Tinnitus	0.94	0.91	0.92	88
Accuracy	-	-	0.94	202
Macro average	0.94	0.93	0.93	202
Weighted average	0.94	0.94	0.94	202

The overall accuracy of the model was 94%, with a macro average and weighted average of 0.94 across precision, recall, and the F1-score. This demonstrates that the ANN model is competitive with classical models, offering a deep learning approach to tinnitus diagnosis with high-frequency audiometry data. Additionally, the performance of the proposed ANN model was assessed using cross-validation with KFold, yielding a result of 90.3%. This cross-validation performance confirms the model's robustness and generalizability across different subsets of data.

**Figure 6:**
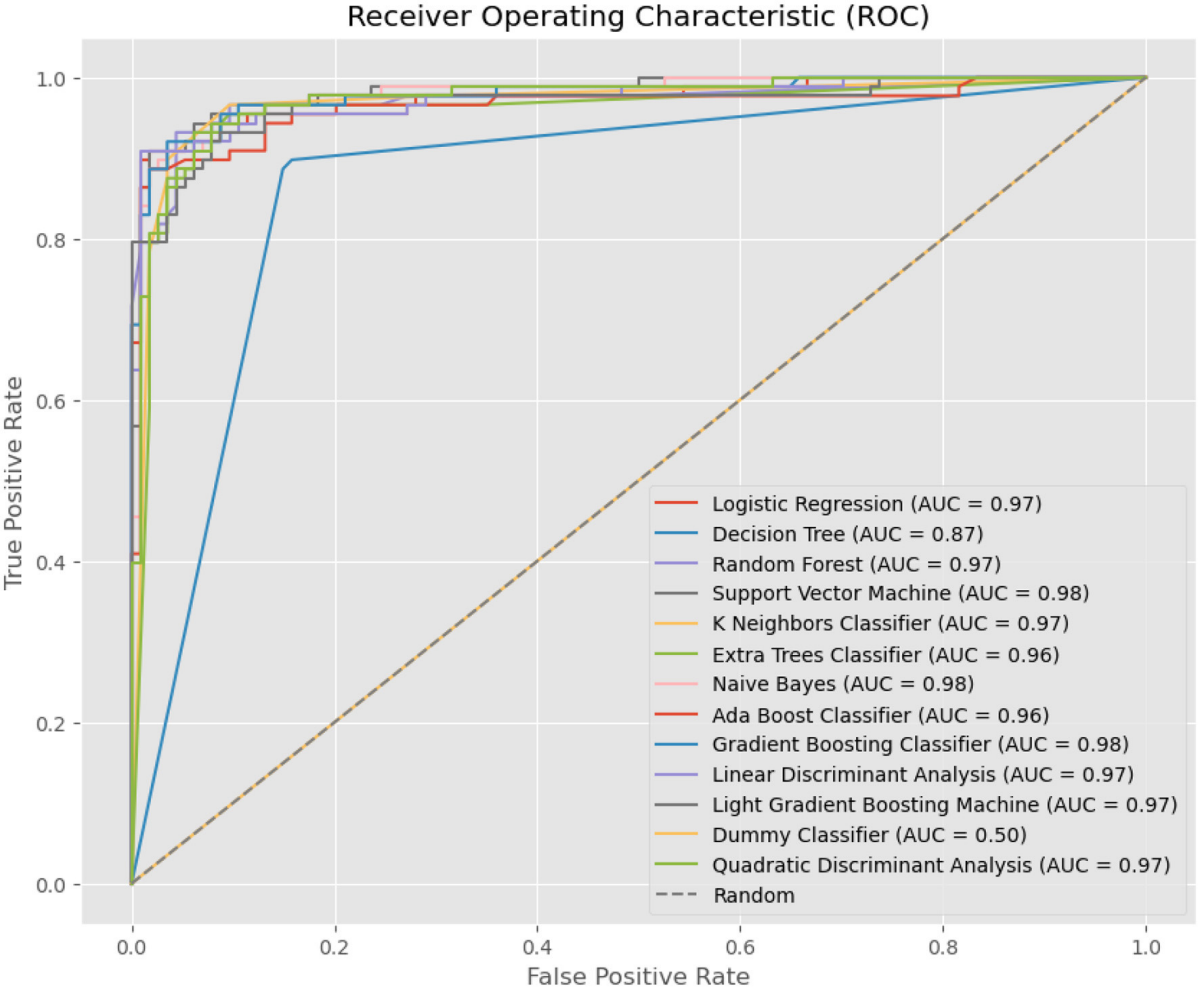
ROC Curves for Machine Learning Models in Tinnitus Diagnosis, showing the True Positive Rate vs. False Positive Rate and the AUC for each model, highlighting their diagnostic accuracy.

**Figure 7:**
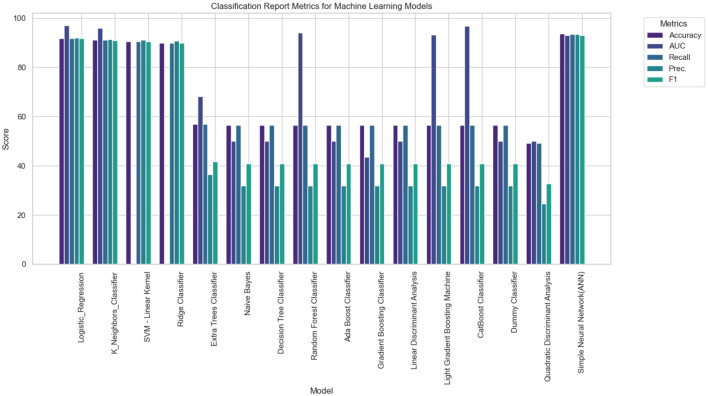
Performance Evaluation of Various Machine Learning Models for Tinnitus Classification, showcasing accuracy, AUC, recall, precision, and F1 score for each algorithm.

**Figure 8:**
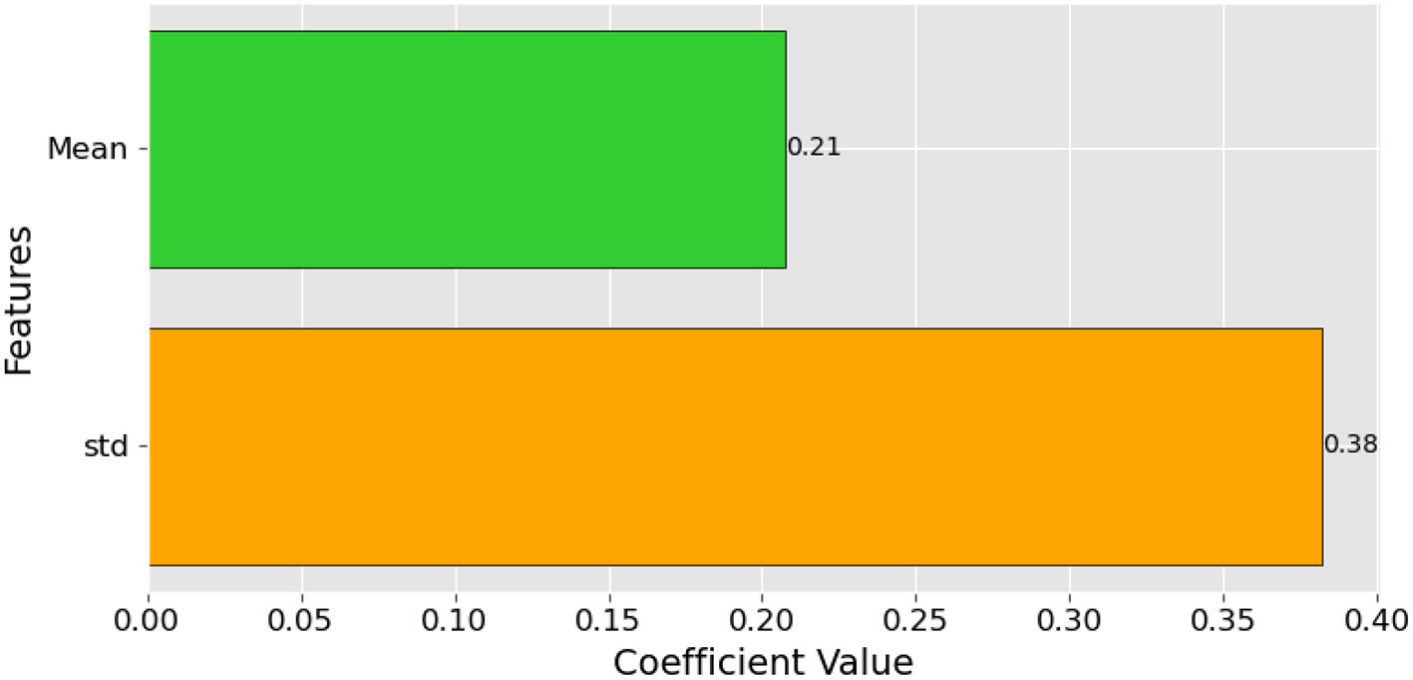
Feature Importance Scores for the Logistic Regression Model in Tinnitus Diagnosis.

Figures 6–8 provides a comprehensive overview of the performance metrics for various machine learning models in classifying tinnitus. Each model is evaluated on six metrics: Accuracy, AUC, Recall, Precision, and F1 Score. The models range from traditional algorithms like LR to more complex ones like a Simple Neural Network (ANN). Across the board, we observe a mixture of performances; however, certain models stand out with particularly high scores in specific metrics. For instance, SVM with Linear Kernel and the Simple Neural Network exhibit high Accuracy and AUC, indicating strong overall performance and the ability to distinguish between classes effectively. On the other hand, models like the Dummy Classifier lag significantly behind in all metrics, serving as a control to indicate the baseline performance level. The variance in height for each metric across models suggests that no single model excels in all areas, emphasizing the need for careful model selection based on the specific performance metric of interest for tinnitus diagnosis.

### 4.4 Feature importance of the LR model

In our LR model for tinnitus diagnosis, the feature importance scores are quantitatively depicted in the accompanying bar chart. It illustrates that 'std', the standard deviation of high-frequency audiometry thresholds, holds the most significant coefficient value of 0.38, suggesting that variability in hearing threshold is a powerful indicator of tinnitus. 'Mean', the average threshold level, with a coefficient value of 0.21, also shows substantial relevance, indicating that average hearing ability at high frequencies is a strong predictor of the condition. These scores provide insight into the key factors contributing to the presence of tinnitus, underlining the importance of both the consistency and variability of hearing thresholds in the clinical assessment of the disorder.

## 5 Discussion

The results obtained from the implementation of various machine learning models, including a LR classifier and an ANN, provide a comprehensive overview of the potential of these algorithms in the diagnosis of tinnitus using high-frequency audiometry data. The LR model demonstrated a high degree of accuracy (91.76%) and an impressive AUC of 97.06%. The high recall (91.76%) and precision (92.04%) indicate that the model is adept at correctly identifying both the presence and absence of tinnitus with few false positives or negatives. These metrics suggest that the LR model is a strong candidate for clinical use, where high sensitivity and specificity are crucial. The ANN model was constructed to compare deep learning techniques with classical machine learning approaches. With an accuracy of 94.06%, the ANN model slightly outperformed the LR in terms of raw accuracy. The classification report indicates high precision and recall for both classes, which translates to high confidence in the model's predictions and its ability to identify true cases of tinnitus.

The ANN's slightly superior performance in accuracy compared to the LR could be attributed to its ability to capture non-linear relationships in the data through its multiple layers and non-linear activation functions. However, the complexity of ANN models can lead to longer training times and may require more computational resources, which is a critical consideration in clinical settings. Additionally, ANN models generally require larger datasets to perform optimally and avoid overfitting. The cross-validation result of 90.3% for the ANN model, while slightly lower than the accuracy obtained on the test set, still indicates a robust model. Cross-validation is essential to ensure that the model generalizes well and is not overly fitted to the training data. Both models demonstrate the potential to enhance diagnostic accuracy for tinnitus, which can lead to better patient outcomes. The high performance of the models suggests that machine learning could be integrated into audiological assessment tools, providing a valuable aid to clinicians in diagnosing tinnitus.

The incorporation of point detection in the analysis of high-frequency audiometry data represents a significant advancement in the diagnostic process for tinnitus. Point detection refers to the identification of specific data points or patterns within the audiometric frequency spectrum that are crucial for the diagnosis of tinnitus. This section discusses the enhancement of diagnostic capabilities through point detection. High-frequency audiometry data contains subtle nuances that standard audiometric evaluations may not capture. Point detection algorithms can identify these subtle changes in the auditory thresholds that may be indicative of early-stage tinnitus or mild cases that would otherwise go unnoticed. This sensitivity is particularly important for a condition like tinnitus, where early detection can lead to more effective management. Tinnitus is often characterized by its presence in specific high-frequency ranges. Point detection allows for the precise identification of frequency-specific hearing loss, which is a common correlate of tinnitus. By focusing on these points, the model can differentiate between tinnitus-related hearing loss and other types of auditory impairments, improving the specificity of the diagnosis. One of the traditional challenges in tinnitus diagnosis is its subjective nature, relying heavily on patient self-report and their perceived severity of the condition. The objectivity that points detection provides allows for a measurable and quantifiable approach to diagnosis, reducing the variability introduced by subjective reporting.

The LR model's ability to incorporate point detection has shown to be predictive of tinnitus presence. This predictive power is not only important for individual diagnosis but also for understanding the progression of tinnitus, as point detection might identify the specific frequencies that are most likely to be affected as the condition develops. From a clinical perspective, the use of point detection in machine learning models translates to more accurate assessments without significantly increasing the diagnostic burden. It enables clinicians to quickly identify tinnitus with confidence, thereby facilitating timely and appropriate therapeutic interventions. The significance of point detection in enhancing tinnitus diagnosis also opens avenues for future research. It suggests that further exploration into more sophisticated point detection algorithms could yield even greater improvements in diagnostic accuracy. Moreover, it sets a precedent for the application of similar methodologies to other auditory conditions where high-frequency data is relevant.

The study, while yielding promising results, is not without limitations that must be acknowledged. These limitations include:

- Data diversity and volume:

The models were trained on a dataset that, although high in quality, may not fully represent the global diversity of tinnitus characteristics. Furthermore, the volume of data available for high-frequency audiometry is relatively limited compared to standard audiometry datasets.

- Model generalizability:

The robust performance of the LR model was validated within the context of this study; however, the generalizability of these findings to other populations or to datasets with different distributions remains to be tested.

- Feature selection and engineering:

The study focused on point detection within high-frequency audiometry data as a novel feature engineering approach. However, the potential exists for the development of additional features that could further enhance model performance.

- Machine learning interpretability:

Although LR is known for its interpretability, the complex nature of machine learning models can make them challenging to interpret in a clinical context, which could limit their acceptance by practitioners.

The results of the study align with the broader findings within the literature, suggesting that machine learning can significantly enhance the diagnostic process for tinnitus. The high accuracy and AUC values achieved by the LR model are consistent with previous studies that have demonstrated the efficacy of machine learning in various audiological applications, particularly in pattern recognition tasks. The findings also support the notion that machine learning models can provide a substantial advancement over traditional tinnitus diagnostic methods, many of which rely heavily on subjective self-report measures and lack the objectivity that machine learning can provide. The study contributes to the literature by specifically focusing on high-frequency audiometry data, an area that is underrepresented in current research. The results suggest that machine learning models, particularly those that can interpret complex patterns in high-frequency data, have significant potential for improving the diagnosis of tinnitus. The limitations observed in the study reinforce the literature's call for comprehensive models that can handle the nuanced nature of tinnitus and the complexity of auditory data.

The discussion highlights the potential of the applied machine learning model while acknowledging that further research is necessary to overcome its limitations and fully realize its clinical potential. It also situates the study within the existing body of literature, confirming the value of machine learning in audiology and emphasizing the novel contribution of utilizing high-frequency audiometry data.

## 6 Conclusion

The research findings indicate that the LR model applied to high-frequency audiometry data for tinnitus diagnosis yielded promising results, achieving a high accuracy of 91.76%, an AUC of 97.06%, and balanced precision and recall scores, surpassing other machine learning classifiers. This approach represents a significant advancement from traditional subjective diagnostic methods, offering a more objective and quantifiable means of diagnosing tinnitus. In clinical settings, these findings are noteworthy as the model's high recall rate reduces the risk of false negatives, ensuring accurate identification of tinnitus patients and appropriate care. Additionally, its precision minimizes false positives, alleviating patient stress and reducing healthcare system burden. Utilizing high-frequency audiometry data, which extends beyond standard hearing tests, could lead to earlier and more precise tinnitus diagnoses, potentially improving patient outcomes. Future research should address limitations by expanding datasets, exploring alternative algorithms and feature engineering, conducting clinical trials, and fostering interdisciplinary collaboration with healthcare professionals. In conclusion, the application of machine learning, especially Logistic Regression, to high-frequency audiometry data holds great potential for enhancing tinnitus diagnosis and patient care, laying the foundation for further development in clinical audiology.

## Data availability statement

The raw data supporting the conclusions of this article will be made available by the authors, without undue reservation.

## Ethics statement

The studies involving human data were approved by the Ethics Committee of Urmia University of Medical Sciences, Iran (IR.UMSU.REC.1402.284). Written informed consent for participation was not required for this retrospective study in accordance with the national legislation and the institutional requirements.

## Author contributions

S-AS-Z: Conceptualization, Data curation, Formal analysis, Funding acquisition, Investigation, Methodology, Project administration, Resources, Software, Supervision, Validation, Visualization, Writing – original draft, Writing – review & editing. AS: Conceptualization, Data curation, Formal analysis, Funding acquisition, Investigation, Methodology, Project administration, Resources, Software, Supervision, Validation, Visualization, Writing – original draft, Writing – review & editing. KK: Conceptualization, Data curation, Formal analysis, Funding acquisition, Investigation, Methodology, Project administration, Resources, Software, Supervision, Validation, Visualization, Writing – original draft, Writing – review & editing. HA: Conceptualization, Data curation, Formal analysis, Funding acquisition, Investigation, Methodology, Project administration, Resources, Software, Supervision, Validation, Visualization, Writing – original draft, Writing – review & editing. EH: Investigation, Methodology, Project administration, Resources, Software, Supervision, Validation, Visualization, Writing – original draft, Writing – review & editing, Conceptualization, Data curation, Formal analysis, Funding acquisition. RH: Conceptualization, Data curation, Formal analysis, Funding acquisition, Investigation, Methodology, Project administration, Resources, Software, Supervision, Validation, Visualization, Writing – original draft, Writing – review & editing. AR: Conceptualization, Data curation, Formal analysis, Funding acquisition, Investigation, Methodology, Project administration, Resources, Software, Supervision, Validation, Visualization, Writing – original draft, Writing – review & editing. SH: Conceptualization, Data curation, Formal analysis, Funding acquisition, Investigation, Methodology, Project administration, Resources, Software, Supervision, Validation, Visualization, Writing – original draft, Writing – review & editing. MB: Conceptualization, Data curation, Formal analysis, Funding acquisition, Investigation, Methodology, Project administration, Resources, Software, Supervision, Validation, Visualization, Writing – original draft, Writing – review & editing. MS: Conceptualization, Data curation, Formal analysis, Funding acquisition, Investigation, Methodology, Project administration, Resources, Software, Supervision, Validation, Visualization, Writing – original draft, Writing – review & editing. SG: Conceptualization, Data curation, Formal analysis, Funding acquisition, Investigation, Methodology, Project administration, Resources, Software, Supervision, Validation, Visualization, Writing – original draft, Writing – review & editing. SS: Conceptualization, Formal analysis, Validation, Writing – review & editing.
